# Topological dynamics of the 2015 South Korea MERS-CoV spread-on-contact networks

**DOI:** 10.1038/s41598-020-61133-9

**Published:** 2020-03-09

**Authors:** Chang Hoon Yang, Hyejin Jung

**Affiliations:** 1 Catholic Kwandong University 24, Beomil-ro 579beon-gil, Gangneung-si, Gangwon-do 25601 Korea; 2 Pusan National University 2, Busandaehak-ro 63beon-gil, Geumjeong-gu, Busan 46241 Korea

**Keywords:** Infectious diseases, Health care

## Abstract

Network analysis to examine infectious contact relations provides an important means to uncover the topologies of individual infectious contact networks. This study aims to investigate the spread of diseases among individuals over contact networks by exploring the 2015 Middle East Respiratory Syndrome (MERS) outbreak in Korea. We present several distinct features of MERS transmission by employing a comprehensive approach in network research to examine both the traced relationship matrix of infected individuals and their bipartite transmission routes among healthcare facilities visited for treatment. The results indicate that a few super-spreaders were more likely to hold certain structural advantages by linking to an exceptional number of other individuals, causing several ongoing transmission events in neighbourhoods without the aid of any intermediary. Thus, the infectious contact network exhibited small-world dynamics characterised by locally clustered contacts exposed to transmission paths via short path lengths. In addition, nosocomial infection analysis shows the pattern of a common-source outbreak followed by secondary person-to-person transmission of the disease. Based on the results, we suggest policy implications related to the redesign of prevention and control strategies against the spread of epidemics.

## Introduction

Transmission of infectious disease through contact among individuals increases the risk of outbreaks with epidemic potential. However, understanding how diseases spread over networks of contacts remains a challenge. In particular, outbreaks of potentially devastating infections, such as SARS (2003), Ebola (2014–2015), and Zika (2015–2016), have shown that the dynamics behind the spread of disease has become more complex, limiting our ability to predict and control epidemics. In this regard, patterns of disease transmission should be used to design specific public health strategies to enhance sustainable capacity while building activities to improve government responses to infectious diseases. Therefore, an analysis of disease dynamics based on the contact patterns can be used to build practical guidance while framing disease prevention and management strategies.

The interpersonal contact patterns of disease transmissions have often been discussed in a network context while modelling epidemics^1–4^. Most potential disease contact takes place in localized communities among individuals occupying a local geographic space around the diseased. If such contacts are repeated within a given period, certain patterns of links will arise. These link patterns can be represented as networks, which show the spread of an infection among individuals. Thus, certain disease dynamics represented in a contact network can be characterised by topologies.

Previous studies on super-spreaders have identified two major types of networks, small-world network^5^ and scale-free network^6^. In the small-world network, a small number of shortcuts are discovered either by randomly connecting the nodes or randomly rewiring the links. From the shortcuts, it can be inferred that the average node length between any two individuals is shortened, thereby making geographic distance a causal factor in epidemic outbreaks^7^. In the small-world network context, thus, it is important to control the super-spreading events to prevent completely new outbreaks^8,9^. In the scale-free network, on the other hand, the number of contacts per individual exhibits a power-law distribution of infection links. The variation in the connectivity distribution of the scale-free network is infinite, because it does not exhibit the threshold phenomenon. Hence, an outbreak can occur at any time^10^. It can be inferred from both networks that the average shortest path length and a small degree of separation are important factors in the epidemic network analysis^11^. Furthermore, the super-spreading characteristic of epidemics has been associated with the spatial proximity of neighbouring nodes in the network^5,12^. Localised transmission of the epidemic is facilitated by high clustering coefficients, because of the close spatial proximity in node connectivity and its influence on their relation. Thus, nodes with a high spatial proximity tend to intensify super-spreading events within clusters, making it easy for the disease to spread locally over the considered population or areas.

It is known that three factors can cause a disproportionately large number of secondary contacts during super-spreading events^13^: host factors (including physiological, behavioural, and immunological factors); viral factors (including virulence and co-infection factors); and environmental factors (including density, failure to recognise the disease, inter-hospital transfers, and airflow dynamics). Among these various factors, previous studies have focused specifically on the behaviour of the host and environmental factors in explaining the outbreak of SARS and MERS^14,15^. It has been established that certain behaviours of the hosts, such as doctor shopping (visiting multiple doctors and facilities), play a critical role in the spread of infectious disease, as multiple visits by the super-spreaders can lead to the contamination of several medical facilities. In addition to the behaviour of the infected individual, a high population density also correlates to a higher number of infections emanating from both the SARS and MERS hosts, because the probability of infection in such a setting tends to be high^15^. Given that the edges in the epidemic network represent physical proximity, a high level of clustering implies that infection occurs locally and spreads rapidly^16^.

The 2015 outbreak of the Middle East Respiratory Syndrome (MERS) in South Korea has been paid much attention as the outbreak was the first and biggest to occur outside Saudi Arabia, where the disease was identified in 2012. It has been known that the human-to-human transmission of MERS-coronavirus (CoV), which is a viral respiratory infection caused by a coronavirus, is relatively limited owing to its lower level of contagiousness. According to Centers for Disease Control and Prevention (CDC)^17^, MERS is thought to be transmitted through respiratory secretions. However, the particular way in which the virus spreads is not fully understood. During the MERS epidemic of 2015, 186 people across 16 healthcare facilities were infected, of whom 39 lost their lives. This biggest outbreak in a relatively short time began with an ‘index patient’, who had visited the Middle East and returned to Korea on May 4. The patient sought treatment for respiratory symptoms at several healthcare facilities and was later confirmed to have a MERS infection on May 20. By then, 31 people had come in contact with the patient, including family members, patients, visitors and hospital staff. In one instance of contact, a second patient was exposed to MERS while sharing an emergency room where the index patient sought care. The two patients became super-spreaders, assumed to generate many transmission events. Thus, they were likely to initiate infection among the susceptible population. Our expectation is that super-spreaders are more likely to hold certain structural advantages in facilitating continued transmission.

This study investigates the spread of infections over networks of contacts among individuals by exploring the 2015 MERS outbreak in Korea. We assume that the spread of a disease in a population depends on both the dynamics of the disease transmission and the structure of the contact networks over which they spread^1,18–24^. One perspective contends that the hosts who transmit the MERS infection are those who are highly central in the contact network. Thus, many neighbouring hosts form relational ties to others vulnerable to infection. Another perspective argues that if a host has already been infected and other hosts are not yet exposed, healthcare facilities play the pertinent role of delivering the infectious virus to other susceptible hosts. We analyze structural network properties of the epidemic transmission by examining both the relationship matrix of the infection tracing of infected individuals (from-whom-to-whom) and the bipartite transmission routes of infected individuals by healthcare facilities visited for treatment. In this study, we explore two research questions about the MERS outbreak in Korea: (a) How did the infectious disease become widespread through a network in a relatively short period of time?; and (b) How did a small fraction of individual hosts spread the MERS virus to a majority of the population?

## Methods

### Data

The dataset contains records of all 186 people, across 43 hospitals, who were infected with the MERS virus between May 20 and July 4, 2015 in Korea. The data is publicly available on press mentions from the Korea Centers for Disease Control & Prevention. The data includes diagnosis and reporting date, sex, age at diagnosis, source of infection, transmission route, and stage of MERS. Scholarly papers in various journals and news media also provided current issues of the MERS event. The use of multiple sources of evidence allows us to validate the research findings. In this study, the contact patterns coming from Marquetoux *et al*.^25^ are established by tracing the relationship matrix of individuals and hospitals. The data for the analysis contains two types of contacts: personal and hospital. A personal contact is defined as the person-to-person route. The format for a personal contact is a daily record. For every pair of individual hosts between whom infectious contacts occurred on a given date, there is a daily record including the source (infector) and target individuals (infected) presented with the spread of the MERS infection. A hospital contact is defined as a hospital-acquired infection, also known as nosocomial infection. A contact occurs when an infected individual visits healthcare facilities for a limited time period for MERS diagnosis, treatment, and follow-up visit records. Each hospital contact corresponds to an actual visit of the individual hosts and includes the date on which the contact event occurred.

We construct a matrix of contact patterns of individuals and hospitals. The number of observations for the personal contacts are 161(source: *n*) and 31(target: *k*), and those for the hospital contacts are 186 (infected host: *n*) and 43(hospital: *k*). Among the 186 confirmed cases (including one case confirmed by China), the rates of infection were confirmed to be as follows: 44.1% (82 cases) among in- or out-patients sharing wards with the MERS patient; 34.4% (64 cases) among the hospitalised MERS patient’s cohabiting family members or visitors; and 21.0% (39 cases) among the healthcare workers or staff employed at the MERS-affected hospital^26^. Based on this data, we use an adjacency matrix to construct contact networks consisting of all recorded transmission routes of MERS infections from May 20 to July 4. In the personal contact network, each pair of individuals, *p*, is examined to determine whether the infection was passed from a source individual *i* to a target individual *j*. Thus, R_*ij:p*_ is equal to 1 if there is an infectious contact between individuals *i* and *j*; the personal contact network is as follows: R_*p*_ = 1 if R_*ij:p*_ = 1 and 0 otherwise. In the hospital contact network, we first use a two-mode incidence matrix to form individual host-by-hospital contact relations, in which R_*ij:h*_ = 1 if individual host *i* visited hospital *j*, and R_*ij:h*_ = 0 otherwise. Next, we construct a bipartite graph consisting of the union of the individual hosts and hospitals as nodes with the edges only connecting individual hosts with hospitals. For hospital-acquired infection, therefore, individual hosts are connected only by co-presence in hospitals, and hospitals are connected only by having individual hosts in common. Thus, the exposed contacts through which the infection can spread are represented by the co-presence of two individual hosts in the same hospitals. All the data in MS Excel, network measures, and visual displays of disease contact relations are produced using UCINET 6^27^, Net-Draw^28^, and VOSviewer^29^.

### Analytical techniques

This study examines the 2015 MERS outbreak in Korea using network analysis tools such as the indicators of centrality, egocentric network, core-periphery, and cut-points. First, centrality pertains to the node’s (as unit of analysis: individual host) position in a network through specific analysis that is preoccupied with the degree, betweenness, closeness, and eigenvector of the geodesic distances^30^. Then, the egocentric network is used to identify the topologies of the contact networks in which single individual hosts are bound with their neighbouring individual hosts to reveal the link between direct contact and the spread of the MERS infection^31^. Furthermore, the core-periphery structures in a given network can reveal how the infection is transmitted locally or globally through the interaction of the core and periphery^32^. Finally, a cut-point approach is used to identify the key hosts whose removal from the network would divide the network into un-connected parts, indicating a potential weakness in the network or disruption of the infection transmission flow^33^. The comprehensive analysis can reveal how a disease contact network can be formed among individuals exposed to nosocomial infections, and the impact of the super-spreaders on the prevalence of infectious diseases within a relatively short period.

## Results

### Network property of small-world

Every node can be reached from every other node by a small number of steps in a small-world network^5^. The personal contact network was assumed to have distinct clusters of individual hosts exposed to the path of MERS transmission, whereas the geodesics among these clusters were small. In a given network of personal contacts, the overall network density of 0.014 implies that only 1.4% of potential personal connections were actually realized. The average path length was 3.131 (disregarding the directness of relationships), and the average clustering coefficient was 0.258, which is significantly larger than the value for the corresponding classical random network, 1.117/162 = ∼0.007 (Table 1). Thus, the network was considered to have a small-world property (small-world index: 1.046) with the presence of a small number of highly connected hub hosts (scale-free characteristics). Additionally, the distance-based cohesion of 0.355 demonstrated a large fragmented network with a relatively weak structural cohesion. That is, the network would become disconnected if specific hosts who acted as shortcuts between the two clusters removed.

**Table 1:** Structural properties of personal contact network.

Category	Value
Network Density	0.014
Average Clustering Coefficient	0.258
Average Path Length	3.131
Distance-based Cohesion (Compactness)	0.355
Small-World Index	1.046
Average Degree	1.117
Total number of Nodes	162

**Figure 1:**
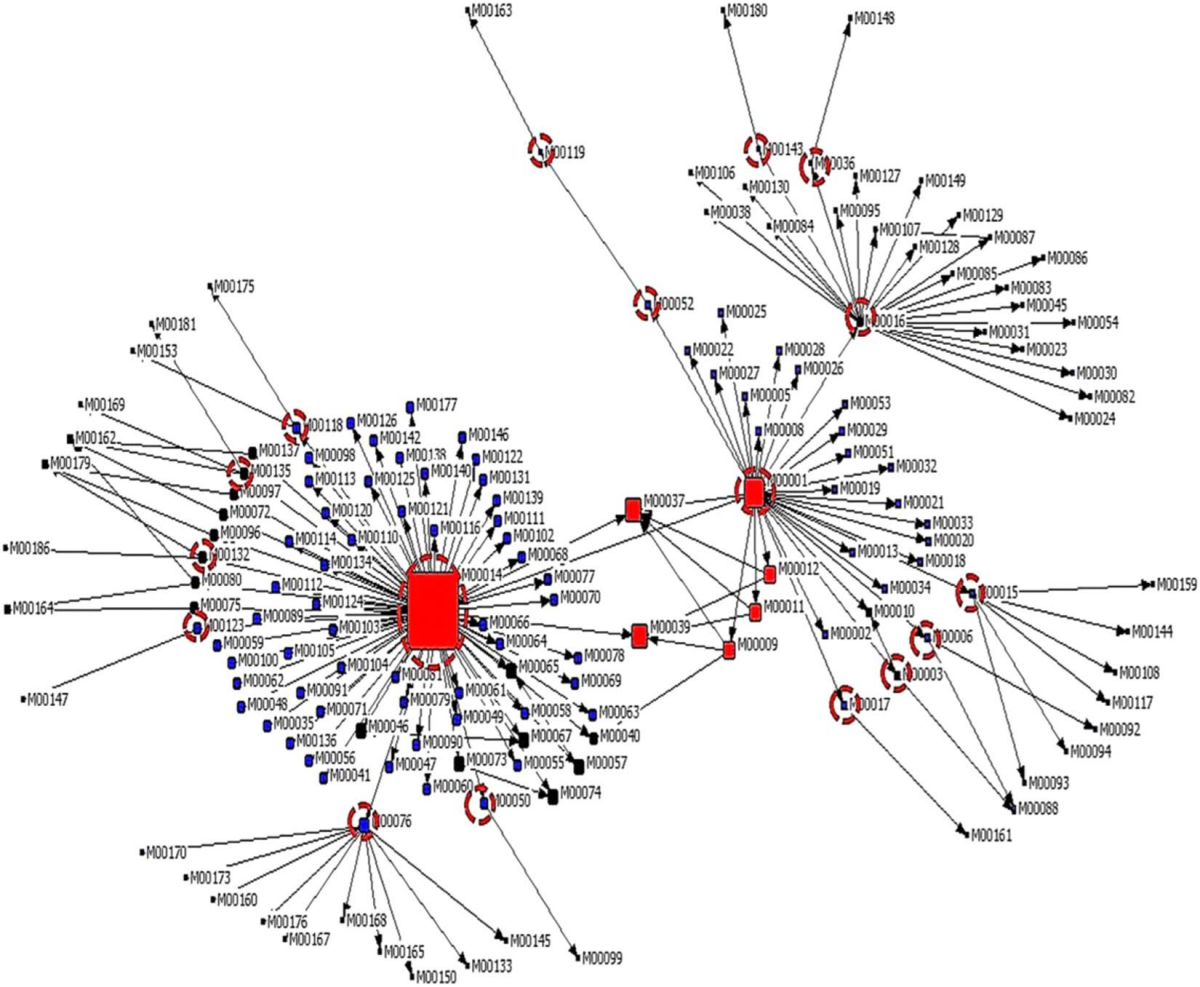
Personal Contact Patterns in MERS Infection Transmission. The overall relations of infection transmission within the personal contact network; three connected sub-structures are identified with the k-core algorithm. A k-core is defined as a hierarchical set of hosts based on a range for each number of contacts they each have according to the degree of connection the hosts have in the network. All nodes represent hosts having contacted MERS infection. Thus, all hosts that generated a given transmission event to *k* other hosts form a sub-structure, and any host that generated multiple transmission events will link multiple sub-structures. Colors correspond to the k-core partition (red: the first, blue: the second, black: the third group) and the size of the nodes in each k-core is proportional to the individual node eigenvector centrality values.

As shown in Fig. 1, the relations of infection transmission within the personal contact network are visually displayed by three sub-groups based on k-core regions^34^. The first group, marked in red, contains hub infectious hosts (#14, #1), as well as the high in-degree susceptible hosts (#37, #39, #9, #11, #12). The second group, marked in blue, comprises susceptible hosts mainly infected through direct contact with hosts #14 and #1. The third group, marked in black, includes the most peripheral indirect host infections through interpersonal transmission (see Supplementary Figs. 1–3 for further details).

### Overall network relationship of personal contacts

In network analysis, the nodes are individual hosts, and the links between individuals represent interactions that may allow disease transmission. The descriptive statistics demonstrate that the average degree of infectious hosts was 1.117, which was quite low, given that there were 161 other hosts (Table 2). We see that the range of out-degree was significantly larger than that of the in-degree (minimum and maximum), and that there was more variability across the hosts in the out-degree than in the in-degree (standard deviations and variances). The coefficient variations for out-degree and in-degree were 5.87 and 0.47, respectively. Thus, the population was more heterogeneous in structural contact positions with regard to out-degree than with regard to in-degree. The overall centralization of out-degree was high at 46%, and the in-degree centralization was low at 2.4% of these theoretical maximums. We arrived at the conclusion that the network of disease transmission might have been dominated more by key hosts than by various groups of individuals. That is, the heterogeneity of the number of contacts tended to affect the spread and persistence of infection^6,25,35^.

**Table 2:** Centrality measure scores and descriptive statistics for the top-5 ranked hosts.

Rank	Degree Centrality				Betweenness Centrality		Closeness Centrality				Eigenvector of geodesic distance	
	OutDeg	Value	InDeg	Value	Bet	Value	OutFar	Value	InFar	Value	Eigen	Value
1	M14	74(45.96)	M37	5(3.11)	M14	91.75(0.356)	M1	314	M162	25119	M14	0.69
2	M1	31(19.26)	M39 M162	4(2.48)	M16	25(0.097)	M14	10968	M37	25277	M1	0.161
3	M16	23(14.29)	M179	3(1.86)	M76	20(0.078)	M16	22059	M39	25278	M37	0.111
4	M76	10(6.21)	M10 M40, M46 M65 M67 M74 M107 M164	2(1.24)	M15	6(0.023)	M76	24472	M179	25280	M39	0.093
5	M15	6(3.73)			M135	4.5(0.017)	M15	25116	M164	25441	M76	0.089
Mean		1.117		1.117		1.086		25751.15		25751.15		0.048
Std. Dev.		6.556		0.526		7.604		2351.923		129.203		0.062
Variance		42.98		0.276		57.818		5531541.5		16693.4		
Minimum		0		0		0		314		25119		
Maximum		74		5		91.75		26082		26082		
Network centralization	45.55%		2.43%		0.35%		47.20%				99.18%	

### Network centrality

We sought to investigate the degree centrality of MERS contact relations, because super-spreaders involved in disease transmission pathways are known for their role in disease dynamics. The degree centrality describes the extent to which an individual host may be cohesive to a network of personal contact relations. In the degree centrality results (Table 2), hosts #14 (M14) and #1 (M1) had higher percentages of out-degree links, indicating that they were in direct infectious contact with many other target hosts in the network, because each had more than 19% network centrality (46% for #14 and 19.3% for #1). Most other hosts in the network had relatively fewer connections. These direct contacts made hosts #1 and #14 more accessible to other hosts, generating several transmission chains that led to the infection of the susceptible target population in the network. This means that small fractions of highly connected ‘hub’ hosts acting as potential super-spreaders played a pertinent role in fueling and driving epidemics of infectious diseases (infectivity)^36–39^. In other words, both host #1 (the index case: the first host infected in a chain of transmission) and host #14 (secondary case: typical infectious host) were well-characterised super-spreaders who held prominent structural advantage in facilitating continued transmission of the infection to susceptible hosts in their neighbourhoods. By contrast, host #37 had a high in-degree and was more at risk of becoming infected through contact with other infectious individuals, followed by hosts #39, #162, and #179. In other words, the more often a host was exposed to a potential threat by way of coming in contact, the more vulnerable he or she was to the risks leading to the MERS infection (vulnerability).

Regarding betweenness centrality, which measures the extent to which a particular individual host lies between other hosts in the network, the overall network centralization was very low at 0.35% of the purely centralized network (Table 2). Thus, most contacts could be made in this network without the aid of any intermediary. Despite this structural constraint, hosts #16 and #76, along with the typical infectious host #14, provided a spatial link or pathway through which MERS was transmitted from the source to the target host populations. For instance, host #14, who acquired the infection through contact with infectious host #1, infected host #76, and the infection transmission continued to emerge through personal contacts within a population of susceptible hosts. Without the tie to bridge hosts, therefore, other susceptible individuals might have been largely isolated from MERS infections.

The closeness centrality indicates the potential independence of a host from the relation of disease contact. With closeness, hosts #1 and #14 had significant potential to make infectious contacts with the target host because of the shorter paths to transmission possibilities, thereby enabling the initial introduction and subsequent spread of MERS towards its target population with transitory contacts. As a result, overall closeness centralization was higher than betweenness centralization. This suggests that there were decentralized short-distance disease contact routes with centralised hub hosts. However, no significant mediation was observed (47.2%: the distributions of in-and out-closenesses could not be computed because the network was weakly connected).

Finally, the eigenvector of geodesic distances offers a measure of the diversity of a personal contact’s network. The results show that host #14 had the highest eigenvector centrality, indicating the consistent hub host of infection transmission in the personal contact network, which is more central to the main pattern of contact distances among all of the individual hosts (degree of inequality was 99.18% of the maximum possible; Table 2).

### Egocentric personal contract network

To understand the modular structures of this disease contact matrix, egocentric personal contact networks were examined. The ego networks of personal contacts consist of both a focal infectious host (*ego*) and a set of susceptible hosts (*alters*), which received infectious contacts from the *ego*, and a measurement of contact relations among these *alters*. The results showed that host #14 was the infectious individual host having the largest egocentric network, and 74 individual hosts had established infectious contacts (Table 3). Among the 74 susceptible hosts in the network of host #14, four were direct person-to-person transmitted infections out of 5,402 possible infectious contact relations, which confirmed the presence of 0.07% of all possible contact relations of infection transmission between hosts. The results of other infectious hosts also show the very fragmented nature of egocentric contact network relationships. Thus, the spread of the MERS infection by hub infectious hosts was more likely in a small-world network, where the individual hosts were not tightly connected in local structures (see Supplementary Fig. 4).

**Table 3:** Egocentric structural metrics for the top-5 highest degree infectious hosts.

Rank	Hosts	Size	Ties	Pairs	Density
1	M14	74	4	5402	0.07
2	M1	31	5	930	0.54
3	M16	23	1	506	0.20
4	M76	10	0	90	0.00
5	M15	6	0	30	0.00

### Analysis of core-periphery structure and cut-point

A core-periphery structure based on the density of connections comprises at least a pair of core and periphery blocks. The core block has many intra-block connections, whereas the peripheral block has relatively few. Contact density was higher for core-to-core relations (0.3). Each core host contacted, on average, 30% of the other hosts in the core of this network and tended to transmit the MERS infection more densely among themselves, representing six identified hosts at a higher risk of infection: high out-degree hosts #1, #9, and #14 with high in-degree hosts #37, #46, and #67 (Table 4). Core hosts were also a source of infection transmission for peripheral hosts (0.108). However, this relation was significantly looser than the one at the core position (core hosts tended not to receive infection from peripheral hosts: 0.002). This shows that hosts in the core transmitted 54 times as many MERS infections to peripheral hosts as peripheral hosts infected core hosts. In other words, the personal contact clusters at the core position via hub hosts were considered to be very dense local structures, where a small number of tightly embedded hosts could cause the MERS infection to spread locally in a population.

**Table 4:** Density matrix for categorical core-periphery model.

	Core*	Periphery
Core (*N*_*c*_ = 6)*	0.3	0.108
Periphery (*N*_*p*_ = 156)	0.002	0.003

*Hosts #1, #9, #14, #37, #46, and #67.

Additionally, a cut-point analysis as an indicator of network’s weak spots vulnerable to disruptions in the infection transmission flow was performed to determine the key hosts who could act as brokers among otherwise disconnected personal contact relations in the network. Around 141 blocks into which cut-points divided the network structure were identified, with 17 cut-point hosts {hosts #1, #3, #6, #14, #15, #16, #17, #36, #50, #52, #76, #118, #119, #123, #132, #135, and #143}. If those 17 hosts were removed from the personal contact relation, the network structure would be fragmented into 141 unconnected parts (red dots in Fig. 1). We see that a number of hub infectious hosts with top-ranked centrality were in cut-point positions, suggesting that they may have been targeted for control strategies to limit the spread of the MERS infection in this population.

### Nosocomial infection

**Figure 2:**
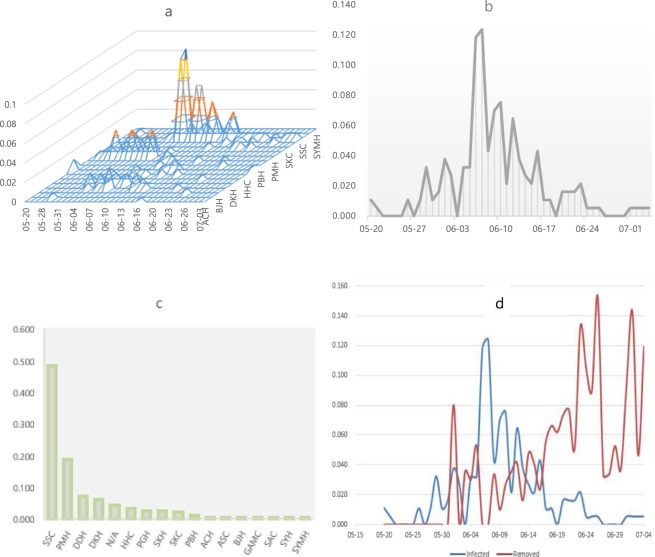
Distribution of MERS introductions as a function of time and place. (**a**), The posterior distribution for both date and place of MERS infection in 16 major healthcare facilities between May and July 2015. (**b**), The posterior distribution for time (date) of MERS introductions. (**c**), The posterior distribution for place (healthcare facilities) of MERS introductions. (**d**), The posterior distribution for MERS introductions and recoveries. *ACH (Asan Choongmoo Hospital, Asan, located in the centralwest of Korea); ASC (Asan Seoul Clinic, Asan); BJH (Joeun Gang-An Hospital, Busan, southeast); DDH (Dae Cheong Hospital, Daejeon, central); DKH (KonYang University Hospital, Daejeon); GAMC (Gangneung Medical Center, Gangneung, east central); HHC (Hallym University Medical Center, Hwaseong, northwest), PBH (Pyeongtaek Bagae Hospital, Pyeongtaek, northwest); PGH (Pyeongtaek Goodmorning Hospital, Pyeongtaek); PMH (Pyeongtaek St. Mary’s Hospital, Pyeongtaek); SAC (Seoul Asan Medical Center, Seoul, northwest); SKC (KonKuk University Medical Center, Seoul); SKH (KyungHee University Hospital at Gangdong, Seoul); SSC (Samsung Medical Center, Seoul); SYH (365 Seoul Yeol Lin Hospital, Seoul); SYMH (Yeoido St. Mary’s Hospital, Seoul).

We identified 43 healthcare facilities where patients sought treatment for respiratory symptoms related to the MERS infection between the months of May and July 2015. We assumed a probability of the introduction of the MERS virus proportional to the daily patient flow of each hospital. We then observed the timeline of the MERS epidemic, as reported by the Ministry of Health and Welfare, and calculated the probability of distributions for the time and place of the introduction of MERS in Korea. Figure 2a shows the posterior distribution for MERS introductions in 16 out of the 43 major healthcare facilities initiating a hospital-acquired outbreak. Figure 2b,c show the generated posterior distribution of the introduction time and place, respectively. These are indicative of a change in the epidemic spreading behaviour of MERS.

The largest posterior distribution was associated with an introduction of hospital-acquired outbreak in SSC (the full name of hospital abbreviations in Fig. 2) on June 7, 2015, and over 60% of infection transmission was more likely to occur between June 6 and 16. The places where MERS was most likely to be introduced were SSC and PMH. Although SSC generated the highest probability of MERS transmission between infected and susceptible individuals, the earliest outbreak was observed in PMH on May 21, which failed to control MERS transmission from the index patient (host #1) to other susceptible neighbours. As shown in Fig. 2d, the epidemic in most healthcare facilities declined after June 7, because the sudden outbreak of MERS produced an epidemic time curve with a sudden onset and a rapid decline. The epidemic curve was suggestive of a mixed outbreak pattern, in which individual hosts were exposed to the common source (or point source) over a short time and spread infection to others via ongoing person-to-person contact with an average incubation period of 6.8 days (range 4–8 days).

### Network centrality of hospital contacts

Analyzing the contact relationships causing nosocomial infection and identifying the underlying network structure of hospital contact patterns are significant in defining how the nosocomial infection was structurally constructed and how the disease was transmitted. We examined network structures representing hospital contact relations weighted by locations of individual hosts and healthcare facilities. Accordingly, the links from infection transmission consisted of two nodes: individual hosts and hospitals they visited for MERS diagnosis and treatment. The exposed contacts through which the infection could spread are represented by the co-presence of two individual hosts in the same hospital. The bipartite graph of all pairs of nodes shows that eight healthcare facilities (SSC, PMH, DDH, DKH, PGH, SKH, HHC, and SKC) were more central than any of the individual hosts on the degree centrality measures (Table 5). For instance, DKH had 14 links to MERS infection and transmission, whereas hosts #1 and #14 had only four and three links, respectively. It also shows that SSC was the most central and was followed by PMH, DDH, and DKH in this order. In other words, SSC, PMH, DDH, and DKH played key roles in the transmission of MERS via individual movements. There was more consistency among the healthcare facility scores, where SSC, PMH, DDH, and DKH had the highest scores across the centrality measures except in the closeness centrality, where DDH and DKH (where other 14 peripheral hosts visited and infected, respectively) were replaced by PGH in which hub host #14 had connection and passed MERS to host #118. As the eigenvector shows in this result, the largest given to SSC (0.996) provided further evidence of the likelihood of increasing the contact event among individual hosts with MERS infection possibilities.

**Table 5:** Centrality measure scores for the top-20 hospital contacts.

	Size	Degree	Betweenness	Closeness	Eigenvector
SSC	95	0.420	0.200	1.214	0.996
PMH	37	0.164	0.131	1.029	0.053
DDH	14	0.062	0.031	0.665	0.001
DKH	14	0.062	0.027	0.662	0.001
PGH	9	0.040	0.019	0.866	0.026
SKH	7	0.031	0.019	0.709	0.023
HHC	7	0.031	0.013	0.638	0.001
SKC	5	0.022	0.009	0.701	0.011
M173	5	0.022	0.009	0.578	0.002
M1	4	0.018	0.040	1.106	0.110
M118	4	0.018	0.016	0.927	0.107
M76	4	0.018	0.023	0.903	0.106
M89	4	0.018	0.007	0.872	0.105
M90	4	0.018	0.007	0.872	0.105
M115	4	0.018	0.007	0.872	0.105
M178	4	0.018	0.011	0.798	0.008
GAMC	4	0.018	0.002	0.679	0.033
M119	4	0.018	0.009	0.530	0.000
M14	3	0.013	0.039	1.110	0.110
M16	3	0.013	0.057	0.821	0.006

**Figure 3:**
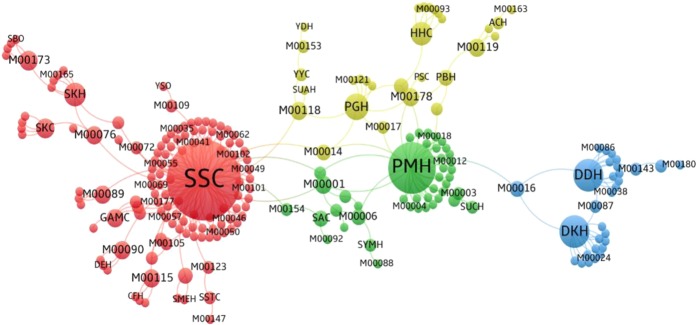
Bipartite graph of MERS infection transmission (hosts-by-hospitals). Structural layout of the network clustered together by different co-occurrence frequency levels of individual hosts and healthcare facilities (small clusters are merged into larger clusters based on modularity function), where red, green and blue nodes denote core nosocomial linkages, and yellow nodes denote periphery linkages. Image was created using VOSviewer.

Looking at the individual hosts from the bipartite method, host #173, who had the highest score compared with that of other hosts, visited five healthcare facilities, four of which were low-centrality hospitals and one (SKH) was a central hospital. It was not as influential as individual hosts such as host #1, who visited two low-centrality hospitals and two central hospitals (SSC, PMH); host #14, who visited just three central hospitals (SSC, PMH, PGH); and host #76, who visited one low-centrality hospital and three central hospitals (SSC, SKH, SKC). In this case, we might expect infectious hub hosts #1 and #14 (along with #76) to have higher centrality scores (i.e., betweenness and closeness) than those of other hosts because they made contact with hosts in the majority of both high-and low-centrality healthcare facilities. We can see the network as comprising four clusters, and these relations were connected by having healthcare facilities in common (for instance, the #1 to #14 relation had the healthcare facility PMH in common; the #14 to #76 relation had SSC in common; the #76 to #173 relation had SKH in common; Fig. 3). This implies that there was a patterned MERS risk for the individual hosts affiliating with these healthcare facilities.

### Core-periphery structure of hospital contact patterns

A core-periphery analysis was conducted to further explore the network structure of hospital contact patterns. The result shows a core composed of 96 individual hosts including high out-degree hosts #1, #16 and #76 with high in-degree hosts #10, #39, #46, #74, and #107, who were all very likely to come in contact with three healthcare facilities (SSC, PMH, and DKH; Table 6). In other words, the core healthcare facilities were more likely to increase the contact event among individual hosts with MERS risk, because they were all visited by 96 or more hosts. The peripheral hospitals had 37 or fewer (partition not shown) hosts. The remainder of the hosts was grouped into the periphery as both presenting less frequently and having few hospitals in common. A considerable number of healthcare facilities were also grouped as peripheral in the sense that they were less visited by infectious hosts, and these hosts had little in common.

**Table 6:** Density matrix for the 2-mode categorical core-periphery model.

	Core(*N*_*cs*_ = 3: SSC, PMH, DKH)	Periphery(*N*_*ps*_ = 40)
Core (*N*_*ca*_ = 96)	0.285	0.016
Periphery (*N*_*pa*_ = 87)^*^	0.240	0.010

^*^Hosts #2, #133, and #145 were exempted from hospital-acquired infections.

On the other hand, a host in the core visited either one or more core hospitals or one core hospital and at least three peripheral hospitals. For instance, nodes that interacted with host #1 were on the core side, whereas nodes that were likely to interact with host #14 were unexpectedly located in the same topological area of the periphery. Host #1 localised in a cluster containing another three healthcare facilities (SSC, PMH, and DKH), which were observed to associate with major nosocomial infections (see red, green, and blue dots in Fig. 3). Interestingly, the SSC cluster was connected via the shared hub host #1 to a cluster consisting of PMH. Additionally, PGH and HHC were present in the periphery and localised to a host #14 partition of the network (yellow dots in Fig. 3). This implies that, based on the idea of the structural equivalence^32,40^, hosts #1 (at the core of the network) and #14 (at the periphery) were not structurally equivalent, although they were highly connected, because they occupied unequal positions or had non-identical relationships with alters. The result also shows that core hosts presented themselves less frequently (0.016) at peripheral healthcare facilities, whereas peripheral hosts presented themselves somewhat more heavily (0.24) at core hospitals (Table 6).

## Discussion

Network analysis tools for exploring the infectious contact network provides a key opportunity to uncover the topologies of the contact networks of individuals in the transmission of MERS. However, the epidemiological topology of the contact network of infectious individuals and healthcare facilities has not, until now, been systematically investigated in relation to MERS. This underscores the necessity of understanding the structural properties in contact networks, particularly as infection transmission correlates to the super-spreading characteristic of epidemics and the prevalence of nosocomial infection in healthcare facilities.

The results show that a small number of healthcare facilities where hub infectious hosts tended to visit for a limited time for MERS diagnosis and treatment had an excessively certain influence on the early spread of MERS throughout the population. The MERS epidemic was more likely to be associated with the increased probability of contact events among individual hosts, and nosocomial infections rapidly increased the proportions of epidemic. Additionally, personal contacts initiated by super-spreaders were considered as potential risk factors for the persistence of the MERS infection on networks and contributed to the epidemic onset with high transmissibility in healthcare settings. In other words, both nosocomial and personal contacts might have played a dominant role in enhancing the risk of the transmission of MERS and subsequent infection in healthcare facilities in a relatively short period of time. This implies that infection prevention and control policies to limit the spread of diseases should aim at targeted surveillance programs and control strategies by investigating and monitoring the introduction and spread of infectious diseases through contact networks.

Our findings contribute to previous studies in several ways. It highlights the role of network analysis tools in analysing the epidemiological topologies of infectious diseases through comprehensive analytical methods. It also underscores the need for further research to develop different epidemiological models for a broader understanding of the structural properties of epidemic transmission. In addition, we reveal that the small-world network with scale-free dynamics is highly relevant to the emergence of complexification of the disease-spreading dynamics, and tends to minimise the average path distance over all the pairs of infectious and susceptible individuals. Finally, notwithstanding the relatively high prevalence and probability of person-to-person disease transmission based on the movement and relationships of individuals, our results confirmed that MERS was often a nosocomial infection.

Based on results, we suggest clear implications on strategy-driven interventions to prevent disease transmission. First, prevention and control efforts should target individuals with the highest likelihood of transmitting the disease. Our results justify the interventions directed towards investigating and monitoring the introduction and subsequent spread of diseases by highly infectious super-spreaders. To mitigate the super-spreading of the MERS among people in homes and in communities, home care for patients with mild symptoms should be provided under close medical observation, after patients and family caregivers must have received appropriate training on personal hygiene, basic infection prevention, and control measures. However, patients with worsening conditions should seek prompt medical attention following a monitor of their health status for 14 days after the exposure event^41,42^. Secondly, our analysis reveals that relatively large tertiary hospitals have higher rates of MERS transmission than small community hospitals, because, in addition to poor disease control facilities, they typically have large numbers of patients and visitors who are engaged in doctor shopping^43,44^. Therefore, it is necessary to put effective quarantine and adequate facility ventilation on the agenda. Furthermore, to improve the response activities of hospital staff in infection prevention and control, timely education and training must be provided^43^. It is also necessary that, regardless of the diagnosis, healthcare facilities should always share essential information in a timely manner with other facilities at the early stage of the outbreak. Finally, until more is understood about MERS, the government should provide reliable and timely information to the public, and establish an efficient disease-control system as preventive measures against the initial spread of MERS.

Despite the comprehensive findings, this study has several limitations. First, it is difficult to control any confounding factors that could influence the study results. Information, such as the determinants of the patient’s choice of healthcare facilities, are frequently not available, which can result in bias in the contact network analysis. Second, this study is based on contact network relationships as traced among individual hosts infected by the MERS. Therefore, it is necessary for future research to identify and analyse the patients in relation to the different patterns associated with both modes of transmission, person-to-person contact transmission and nosocomial infection, to provide a more profound insight into how they differ. Furthermore, the study is limited to the MERS-infected population in Korea. Therefore, the generalisability of findings may be limited. We believe that the limitations of this study can be overcome by comparing various epidemiology models that demonstrate the spread of infections with sufficiently large evidence-based datasets.

## Supplementary information

Supplementary information
