# Supplementary material for: Topological dynamics of the 2015 South Korea MERS-CoV spread-on-contact networks
Source: Sci Rep. 2020 Mar 9;10:4327. doi: 10.1038/s41598-020-61133-9 (PMC7062829; doi:10.1038/s41598-020-61133-9)
Supplement: Supplementary file 1 — Supplementary information [file 41598_2020_61133_MOESM1_ESM.pdf]

Topological dynamics of the 2015 South Korea MERS-CoV spread-on-contact networks

Chang Hoon Yang (Catholic Kwandong University)

24, Beomil-ro 579beon-gil, Gangneung-si, Gangwon-do, Korea, 25601

cy8064@cku.ac.kr

Hyejin Jung*(Pusan National University)

*Corresponding author:

2, Busandaehak-ro 63beon-gil, Geumjeong-gu, Busan, Korea, 46241,

hjung@pusan.ac.kr

Work telephone: 82-51-510-2108

Work fax: 82-51-583-0801

[Supplementary figures: Figs. 1-4]

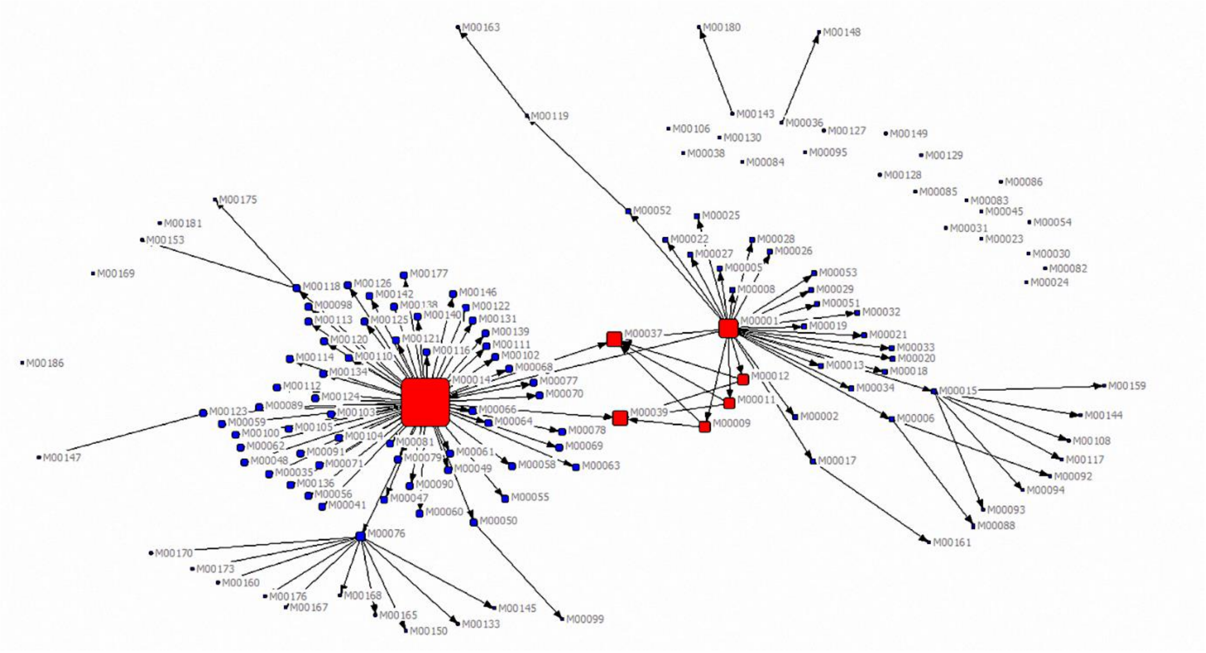

Supplementary Fig. 1 A k-core subnetwork was created removing peripheral hosts of the third group.

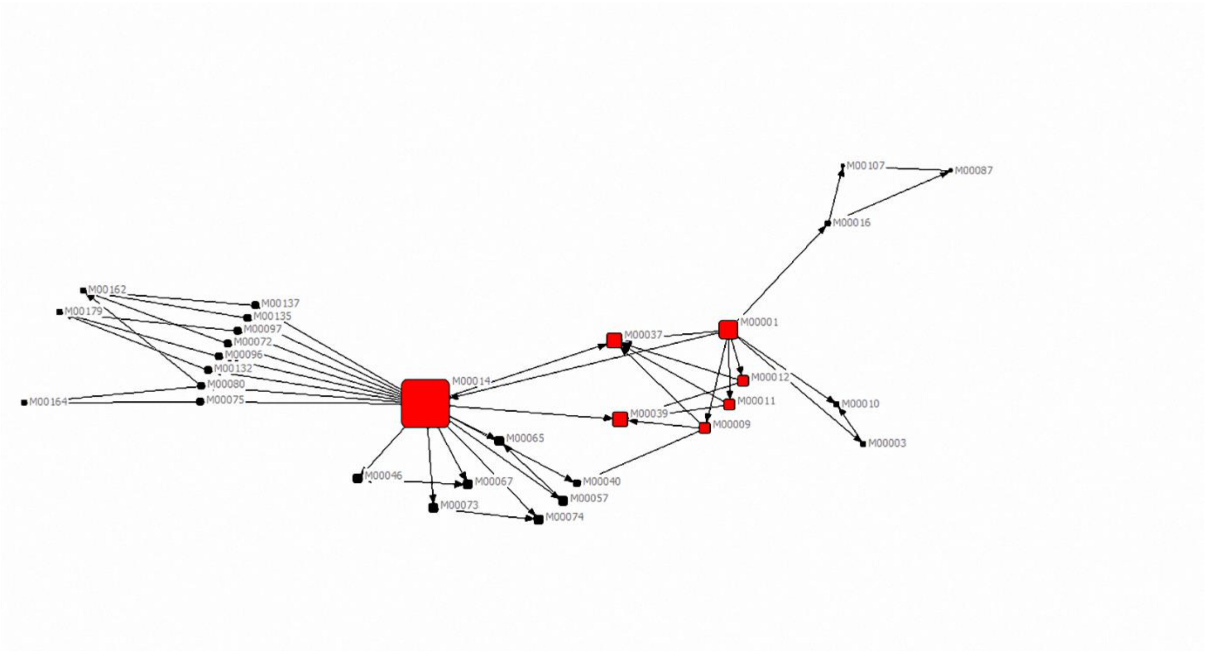

Supplementary Fig. 2 A k-core subnetwork removing susceptible hosts of the second group directly infected by hub hosts #1 and #14.

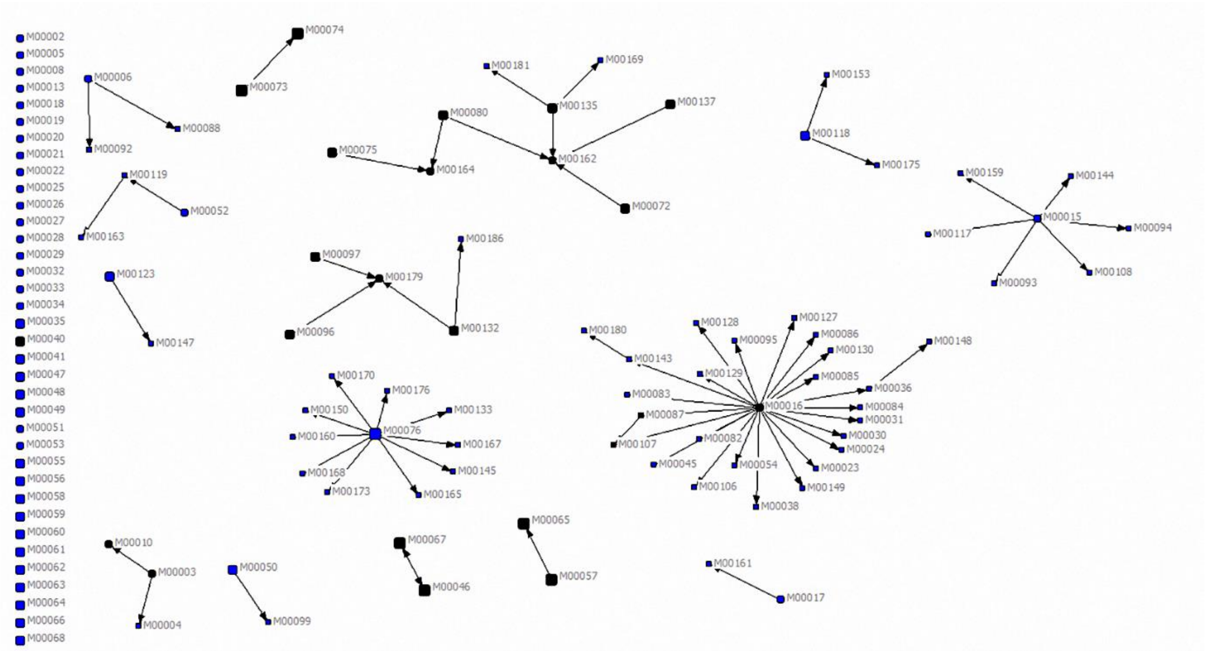

Supplementary Fig. 3 A k-core subnetwork removing high-degree hosts of the first group.

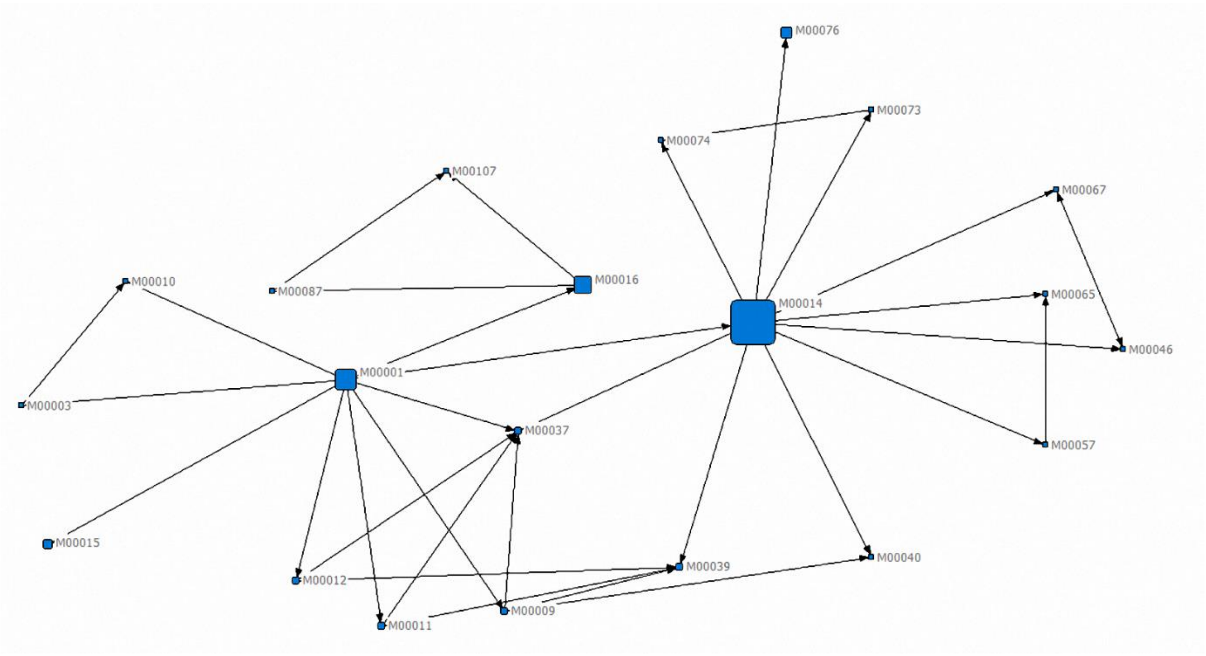

Supplementary Fig. 4 A sample from the egocentric network showing single-level network relationships of host #14 with other four highest degree infectious hosts (#1, #15, #16, #76).
